# Opioid use in postoperative pain management of pediatric appendectomy patients in Japan

**DOI:** 10.1007/s00540-025-03525-7

**Published:** 2025-06-14

**Authors:** Julia Smith Cavalcante, Susumu Kunisawa, Kiyohide Fushimi, Karin Kato, Yuichi Imanaka

**Affiliations:** 1 Department of Healthcare Economics and Quality Management, Graduate School of Medicine, Kyoto University, Yoshida Konoe-cho, Sakyo-ku, Kyoto, 606-8501 Japan; 2 Department of Health Policy and Informatics, Graduate School of Medical and Dental Sciences, Institute of Science Tokyo, Tokyo, Japan; 3 Department of Patient Safety Unit, Kyoto University Hospital, Kyoto, Japan; 4 Department of Health Security System, Center for Health Security, Graduate School of Medicine, Kyoto University, Kyoto, Japan

**Keywords:** Opioid analgesic, Postoperative pain management, Pediatric surgery, Appendectomy, Japan

## Abstract

**Purpose:**

To evaluate the use of opioid analgesics for postoperative pain management in pediatric appendectomy patients in Japan, investigating individual and facility-related factors using hospital administrative data.

**Methods:**

This study was a retrospective cohort study. Data was sourced from the Diagnosis Procedure Combination database. Inclusion criteria were: patients 2–18 years old; admitted and discharged between April 1st, 2018 and March 31, 2020; diagnosed with acute appendicitis; and who underwent appendectomy. The outcome was postoperative opioid use, calculated as the proportion of patients who used opioid from the day after surgery.

**Results:**

There were 11,346 cases that were selected for the study. The overall proportion of patients who were administered postoperative opioids (POAP) was 9.47%. The POAP was similar across ages, sexes and types of surgery, but higher for patients with longer length of stay (LOS) and patients with abscess. The group that was administered opioids preoperatively had higher POAP. Inter-hospital variation was observed, as less than 40% of the hospitals were responsible for all patients who used postoperative opioids. Opioids were prescribed upon discharge to 0.01% patients.

**Conclusion:**

The POAP in pediatric appendectomy patients in Japan was lower than that observed in other countries, which might be attributed to legislation and opioid prescription culture. Hospital variation was observed, which might be related to the lack of guidelines and evidence-based recommendations, and to physician and hospital preferences. Future research is necessary for the development of guidelines that detail the appropriate use of opioids and avoid unnecessary exposure to pediatric patients.

## Introduction

Opioids are commonly used in surgery, both as an analgesic in pre- and postoperative pain management and as part of anesthesia protocols during operation [1]. However, their use carries many risks, such as respiratory depression, postoperative nausea and vomiting (PONV), and inflammation [2]. There have also been reports of higher pain levels after opioid use, which may further consumption [3, 4] and lead to opioid use disorder (OUD) and addiction. Since the exposure to opioids in surgical care and extensive prescription of opioids upon discharge may lead to future opioid misuse [4, 5], there is particular concern for early exposure of pediatric surgery patients.

Appendicitis is one of the most common surgical emergencies in the world, with 17.7 million estimated cases of acute appendicitis in 2019 worldwide, most frequently between the ages of 10 and 20 years [6]. Treatment guidelines focus on early surgical removal of the appendix, or appendectomy [6, 7], and despite research indicating that opioids might not be necessary for postoperative pain management in this surgery [8], guidelines still include them in the first pain relief measures [9]. Therefore, appendectomy may be the first contact with opioids for children and teenagers [10].

Previous studies in the use of opioids in pediatric appendectomy have been conducted in the USA and Australia, attempting to understand some of the factors that can predict the use of opioids. In the USA, Mahdi et al. [11] and Ferguson et al. [12] found that 77.6% and 28% of patients, respectively, used postoperative opioids, showing vastly different usage rates. The former observed that postoperative opioid use was associated with an increased postoperative length of stay (pLOS), and the latter found that the preoperative opioid use was associated with increased postoperative use. Sonderman et al. [13] observed that 68% of patients received prescription opioids upon discharge. In Australia, Ousley et al. [14] observed 99% of postoperative opioid use. Correlations between opioid use and disease severity, as well as differences in the likelihood of postoperative opioid use based on the administration of other analgesic medications were also observed [12, 14].

In Japan, opioids are divided into two groups: narcotic opioids, such as morphine, codeine, and fentanyl; and non-narcotic opioids, such as tramadol and buprenorphine. Usage of opioids is limited by laws that restrict prescribing, especially for the narcotic category [15]; however, opioid misuse and aberrant opioid prescription have been observed [16]. Despite this misuse being more associated with cancer and chronic pain treatment, it is important to take precautions to avoid unnecessary opioid exposure [4, 5]. The evaluation of the opioid usage pattern in pediatric appendectomy and possible related factors in Japan may become an important resource in pinpointing gaps that might need improvement and creating opioid use guidelines for pediatric appendectomy postoperative pain management in the future.

The objective of this study is to evaluate the possible patient and facility-related factors that may be associated with opioid usage in the postoperative pain management of pediatric appendectomy in Japan.

## Methods

### Data sources

This is a retrospective cohort study using Diagnosis Procedure Combination (DPC) data, from the DPC Research Group database, which is funded by the Ministry of Health, Labor and Welfare (MHLW). DPC data comprises claims of inpatients and their information of discharge summaries, including age, sex, LOS, medications prescribed during hospitalization, and International Classification of Diseases, Tenth Revision (ICD-10) codes, which are used to indicate the main diagnosis, cause of admission, most and second most medical resource-intensive diagnoses, up to ten comorbidities, and ten complications [17]. DPC data is generated as anonymized data at each hospital.

### Study population, and inclusion and exclusion criteria

The eligibility for participation in the study was patients 2 years old or older and younger than 18 years old; admitted and discharged between April 1, 2018 and March 31, 2020; diagnosed with acute appendicitis (ICD-10 K35 code was present either as main diagnosis, most or second most medical resource-intensive diagnoses) and who underwent appendectomy (surgery codes K7181, K7182, K718-21, K718-22).

Exclusion criteria included the following: a. patients with multiple surgeries in the same hospitalization period; b. patients with multiple hospitalizations for the same surgery code in the specified admission/discharge period; c. absence of drug administration data; d. participation in clinical trials; f. pregnancy; g. patients with confirmed cancer; h. missing facility data; and i. patients coded as having had no anesthesia during the surgery. The exclusion criteria were selected to remove patients who had missing data or other factors that could influence the use of pain medication.

### Outcomes, variables, and definitions

The primary outcome was postoperative opioid use, represented by the proportion of patients who were administered any opioids at least once on the first day after surgery or on subsequent days, while still hospitalized. This proportion will be referred to as POAP (postoperative opioid administration proportion) for ease of discussion. The proportion of patients who received a prescription of opioids upon discharge was a secondary outcome.

The patient-level variables selected were age (separated in tiers of 2–7, 8–13, 14–17), sex, type of surgical procedure (surgery code: open, K7181 and K7182 or laparoscopic, K718-21 and K718-22), disease severity, as indicated by the presence of abscess during surgery (surgery code: without abscess, K7181 and K718-21, with abscess, K7182 and K718-22), length of stay (LOS) and postoperative length of stay (the length of hospital stay after surgery in days, with the day of surgery as *D* = 0, pLOS), and anesthesia (use of local anesthetics or not; use of epidural anesthesia or not).

Among medication-related variables, use of opioids during preoperative and intraoperative periods and use of other analgesic medications (namely acetaminophen, loxoprofen, flurbiprofen, celecoxib, diclofenac, and ibuprofen) during the perioperative period were included. Preoperative use of any medication was defined as administration before the day of the surgery. Intraoperative use was defined as administration on the day of surgery. Postoperative use was defined as administration on or after the first day after surgery.

Classification of opioid medication used was also included. Opioids were divided into narcotic and non-narcotic as per the Japanese classification. Narcotic opioids included morphine, hydromorphone, oxycodone, codeine, cocaine, fentanyl, remifentanil (analyzed only as anesthetic before and during surgery), pethidine, and methadone. Non-narcotic opioids included tramadol, buprenorphine, pentazocine, and eptazocine.

Facility-level variables included the number of permitted general beds of each facility, which was used to classify facilities per size (facilities with 20–199 beds were considered small, 200–399 were medium, and 400 beds and over were large facilities); case volumes and location of the facility (among the 47 prefectures and 8 regions of Japan). To reflect the differences in the number of facilities in each prefecture, the POAP in each prefecture was calculated as a mean, summing the POAP of the facilities and dividing it by the number of facilities in a prefecture, and will thus be called “mean POAP” (mPOAP) during the discussion. The same was done regarding POAP by region, summing the mPOAP of each prefecture and dividing by the number of prefectures in a given region. Added to the evaluation of the POAP in each facility, the proportion of facilities that administered postoperative opioids to any patient (POAP-F) was also measured.

### Ethical considerations

The study protocol was approved by the Ethics Committee of Kyoto University Graduate School of Medicine, Kyoto, Japan (R0135). The research was conducted in accordance with the Ethical Guidelines for Medical and Biological Research Involving Human Subjects published by the Japanese Government.

## Results

There were 11,722 patients diagnosed with appendicitis that met the inclusion criteria. After screening with the exclusion criteria, 11,346 pediatric patients who had been hospitalized in 892 hospitals were selected for the study.

Out of the selected patients, 9.47% (*n* = 1074) used opioids postoperatively, with 11.4% (*n* = 122) of them using narcotic opioids only, 87.2% (*n* = 937) of them using non-narcotic opioids only and 1.4% (*n* = 15) of them using both. The opioids administered postoperatively for pain management were pentazocine (*n* = 892), fentanyl (*n* = 135), tramadol (*n* = 28), buprenorphine (*n* = 21), eptazocine (*n* = 14), tramadol/acetaminophen (*n* = 12), morphine (*n* = 1), and pethidine (*n* = 1). Prescription of opioids upon discharge was observed in 0.01% (*n* = 12) of patients.

Clinical characteristics of patients are summarized in Table 1. Patients were grouped into those who did and did not use opioids postoperatively, and the proportion of patients who were administered opioids postoperatively (POAP) was calculated. The POAP remained similar for all age groups, both sexes and surgery types, and did not show differences whether the patient was administered local or epidural anesthesia or not. However, it was over double for patients with abscess compared to those without abscess; and an increase in POAP can be seen from the first to the last quartile of both LOS and pLOS, with the 4th quartile’s showing 248% and 319% increase when compared to the 1st, respectively.

**Table 1:** Distribution of patients according to their clinical characteristics and postoperative opioid use

		Total (*n* = 11,346)	*No (*n* = 10,272)	†Yes (*n* = 1074)	‡POAP
Age (y), Mean ± SD		12 ± 3.36	12 ± 3.33	12 ± 3.55	–
	2–7, *n*	1209	1085	124	0.103
	8–13, *n*	5790	5308	482	0.083
	14–17, *n*	4347	3879	468	0.108
Sex, *n*	Male	7037	6372	665	0.095
	Female	4309	3900	409	0.095
Type of surgery, *n*	Laparoscopic	9979	9023	956	0.096
	Open	1367	1249	118	0.086
Severity, *n*	No abscess	8720	8052	668	0.077
	With abscess	2626	2220	406	0.155
LOS (d), mean ± SD		5 ± 2.94	5 ± 2.75	7 ± 4.03	–
	Quartile, *n*	Q1	2699	138	0.049
		Q2	2664	173	0.061
		Q3	2557	279	0.098
		Q4	2352	484	0.171
pLOS (d), mean ± SD		4 ± 2.7	4 ± 2.5	5 ± 3.8	–
	Quartile, *n*	Q1	2720	117	0.041
		Q2	2648	189	0.067
		Q3	2555	281	0.099
		Q4	2349	487	0.172
Local anesthesia, *n*	Yes	9541	8664	877	0.091
	No	1805	1608	197	0.109
Epidural anesthesia, *n*	Yes	293	264	29	0.098
	No	11,053	10,008	1045	0.094

*No means the patient did not use postoperative opioids

†Yes means the patient used postoperative opioids

‡ [POAP] is the postoperative opioid administration proportion, or the proportion of patients administered postoperative opioids

Concerning preoperative pain management, most patients were not administered any of the pain medications evaluated in this study (85.7%, *n* = 10,053) on the day before surgery, and the most frequently administered preoperative pain medication was acetaminophen, in 9.4% (*n* = 1072) of patients (Table 2), with the POAP being similar for both groups. On the other hand, preoperative administration of any opioid was observed in only 1.1% (*n* = 129) of patients, but the POAP for this group was the second highest. The highest POAP observed was for the group administered preoperative non-narcotic opioids, which was over double that of patients administered preoperative narcotic opioids and over four times higher than patients who did not receive any opioids. The POAP was also higher for patients who used flurbiprofen, over double that of the group administered acetaminophen.

**Table 2:** Perioperative use of other analgesic medication and the corresponding postoperative opioid use

Medication	*Preoperative use		*Use on the day of surgery		*Postoperative use	
	†Patients, *n*	‡POAP	†Patients, *n*	‡POAP	†Patients, *n*	‡POAP
Acetaminophen	1072	0.118	9757	0.093	8089	0.098
Loxoprofen	53	0.094	373	0.059	2102	0.100
Flurbiprofen	97	0.247	3190	0.082	1680	0.120
Celecoxib	1	0.000	39	0.077	282	0.145
Diclofenac	82	0.134	1057	0.054	505	0.119
Ibuprofen	4	0.000	31	0.065	83	0.108
Opioids (general)	129	0.372	11,161	0.095	1074	1.000
Narcotic opioid	13	0.154	11,004	0.095	137	1.000
Non-narcotic opioid	116	0.397	1722	0.276	952	1.000
No opioid	11,217	0.091	185	0.086	10,272	-----
No medication	10,053	0.090	11	0.189	1636	-----

*The sum of the columns is not the total of patients, as patients may use multiple analgesics or no analgesic at all

^†^Patients, n refers to the number of patients who were administered that medication at that perioperative time

^‡^ POAP is the postoperative opioid administration proportion, or the proportion of patients administered postoperative opioids

Contrary to the days before the surgery, use of opioids on the day of surgery was observed in 98.4% (*n* = 11,161) patients, and use of non-opioid pain medication also increased, with less than 0.1% (*n* = 11) patients using no medication. The POAP was similar for both the groups that were and were not administered opioids on the day of the surgery; however, the POAP for the group that did not take any medication was double that of the group that was administered opioids. The highest POAP was observed in the group that used non-narcotic opioid on the day of surgery, almost three times the POAP of the group that was administered narcotic opioids. The lowest POAPs were from the groups that were administered diclofenac or loxoprofen.

Postoperatively, the use of pain medication was higher, but there was decrease in the consumption of opioid medication. The POAP was similar for all patients who used other non-opioid medications, with the highest POAP being for the group that was administered celecoxib and the lowest for the group that was administered acetaminophen.

Regarding the administration of opioids from the perspective of the facilities where the patients were hospitalized, the proportion of facilities that administered postoperative opioids to at least one patient (POAP-F) was 38.2% (*n* = 341). Most facilities were large and medium sized (41.8% and 39.8%, respectively). The case volumes, POAP and POAP-F increased with the size of the hospital (Table 3). The POAP-F of large facilities was almost triple that of small facilities.

**Table 3:** Distribution of hospitals, patients, and proportion of postoperative opioid use per hospital size

Hospital size	Case volume*	Facilities, *n* (%)	POAP-F†	Patients, *n* (%)	POAP‡
All	16.4	892	0.383	11,346	0.095
Small	13.0	164(18.4)	0.177	553(4.9)	0.069
Medium	15.0	355(39.8)	0.355	3204(28.2)	0.086
Large	19.3	373(41.8)	0.499	7589(66.9)	0.100

*Case volume is the average annual case volume for each category

†[POAP-F] is the postoperative opioid administration proportion in regard to facilities, or the proportion of facilities that administered postoperative opioids to at least one patient

‡[POAP] is the postoperative opioid administration proportion, or the proportion of patients administered postoperative opioids

**Fig. 1:**
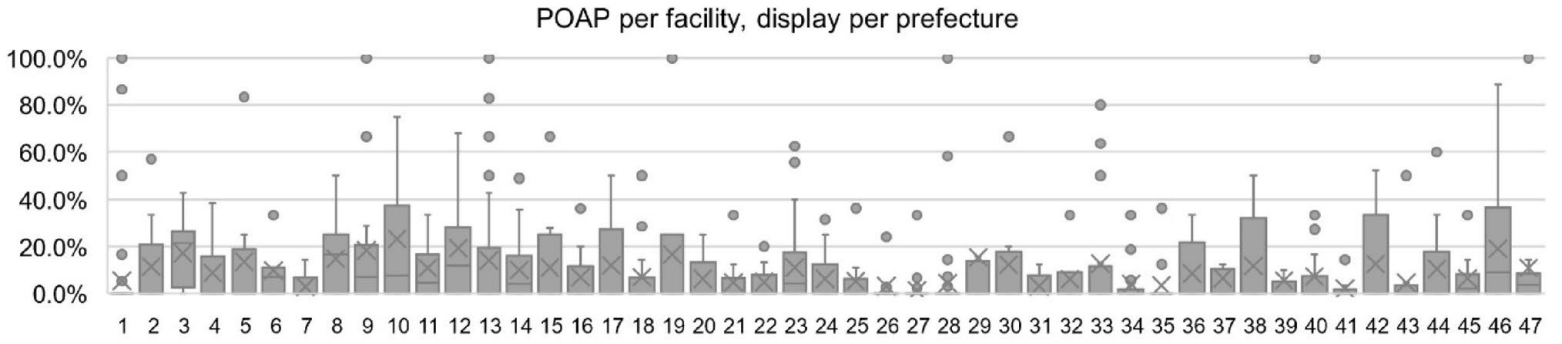
Postoperative opioid administration proportion (POAP) per facility, display by prefecture. Each point corresponds to the POAP of a facility within the prefecture. Prefectures are represented using their corresponding numbers. Dots (●) indicate outliers, (✖) indicates mean, and lines (▬) indicate median

The majority of facilities and patients included in this study were from the regions of Kanto (22.9% hospitals, 25.8% patients), Kansai (18.2% hospitals, 15.6% patients), Kyushu (17.4% hospitals, 17.8% patients), and Chubu (16.8% hospitals, 19.9% patients). Kanto region also had the highest mPOAP, over double that of Kansai (15.8% versus 6.8%) and almost three times that of Hokkaido, the region with the lowest mPOAP (5.4%) (Table 4). The prefectures with the highest mPOAP were Gunma (23.2%), Chiba (19.4%), and Kagoshima (19.1%) with Tokyo taking the ninth place (14.1%) and the lowest mPOAP being from Osaka (1.2%) (Fig. 1).

**Table 4:** Distribution of hospitals, patients, and postoperative opioid use proportions across regions

Region	Number of hospitals, *n* (%)	Number of patients, *n* (%)	mPOAP*
All	892	11,346	9.5%
Hokkaido	52 (5.8)	451 (4.0)	5.4%
Tohoku	71 (8.0)	837 (7.4)	10.6%
Kanto	204 (22.9)	2931 (25.8)	15.8%
Chubu	150 (16.8)	2254 (19.9)	8.9%
Kansai	162 (18.2)	1769 (15.6)	6.8%
Chugoku	65 (7.3)	786 (6.9)	5.9%
Shikoku	33 (3.7)	295 (2.6)	8.1%
Kyushu	155 (17.4)	2023 (17.8)	9.1%

*[mPOA] the mean proportion of patients administered postoperative opioid in each region; calculated by summing the average POAP of the prefectures in a region and dividing by the number of prefectures in that region

Eleven (1.23%) hospitals administered postoperative opioids to all their patients. These facilities only had up to three cases and were distributed evenly across size categories, three being small, four medium, and four large sizes. They were all from different prefectures, one from Hokkaido, five from Kanto, one from Chubu, two from Kansai, and two from Kyushu regions.

Considering the possibility that facilities with less patients presented higher POAP, we investigated facilities with less than ten patients (hereby cases < 10) and facilities with at least ten patients (hereby cases ≥ 10). Considering only facilities with cases < 10 (*n* = 553, 62%), only 24.4% (*n* = 135) of these facilities administered opioids to at least one patient. This facility group had normal distribution of size, being mostly medium sized, and had an average POAP of 9.0%. On the other hand, considering facilities with cases ≥ 10 (*n* = 339, 38%), 60.8% of them (*n* = 206) administered postoperative opioids to at least one patient. These facilities were mostly large sized, with very few representatives of small facilities and had average POAP of 8.7%.

## Discussion

The proportion of pediatric appendectomy patients who used postoperative opioids observed in our study was 9.47%, at most a third of the percentage of patients found to use opioids in the USA [11, 12], showing that Japan has a particularly infrequent postoperative opioid use in appendectomy. This is coherent with the findings of other studies which also found less prescription in Japan as compared to other countries, particularly for acute pain [15, 18].

The reasoning behind the lower use of opioids in Japan is a mix of the strong legislation existing in the country, the first of Asia to regulate and control opioid use [18], as well as the culture regarding opioid prescription and consumption. When it comes to the regulatory side, only physicians can prescribe opioids, differently from countries like the USA, where nurse practitioners and other medical staff can also do so. Also, both the physician and the facilities must hold a special license to be able to prescribe narcotic opioids [18]. The way opioid medication enters the country is also very strictly regulated by the Narcotic and Psychotropic Control Act, which makes it difficult to have contraband [15, 18, 19].

The prescribing and consumption culture is also not favorable for a high use of opioids. In Japan, prescription of strong opioids is done mainly to treat cancer pain, and is not generally recommended for chronic non-cancer pain as a first option [15, 18, 20, 21]. Physicians in Japan also avoid opioid-related side effects by restricting opioid consumption. In addition, the use of opioids is seen badly by the Japanese population, discouraging patients to seek treatment with opioids themselves, and the national health insurance only covers the use of opioids for certain treatments [15, 18]. Finally, Japan has a very low rate of drug abuse, and opioids are not usually the drug of choice for such cases [22].

The regulations mentioned above also help explain the difference in drugs used. The drug with the highest postoperative use in this study was pentazocine, followed by fentanyl and tramadol, with higher use of non-narcotic opioids, which do not require a prescribing license and face less regulation. This is a change from what is seen in the USA and Australia, with studies reporting postoperative use of morphine, fentanyl, oxycodone, codeine, and hydrocodone [13, 14], and this difference is consistent with the findings of Tannoury [20].

Considering both the differences in prescribing numbers and types of opioids used, as well as differences in prescribing and consumption culture, it is important to note that for Japan, following the guidelines established by countries with higher opioid use might offer some risks of increasing opioid consumption. The future development of any recommendation or guideline for opioid use in postoperative pain management of pediatric surgeries must be culturally sensitive and culturally appropriate based on the circumstances of the country to which it will be applied.

Among clinical factors, over double the proportion of postoperative opioid use was observed in patients who had abscess when compared to those without abscess, a finding that was also observed by Mahdi et al. [11]. As an indicator of disease severity and pain, abscesses would justify the higher use of stronger medication for pain. An increase in postoperative opioid use proportion was also observed with the increase in LOS and pLOS quartiles. This can also be related to higher levels of pain, which would force the patient to stay longer in the hospital and justify the use of stronger medication. Decrease in hospital stay has also been associated with fewer opioid use in other studies [11]. Other than this, the proportion of use remained similar in age tiers, age means, sex, type of surgery, and use of local and epidural anesthesia.

The LOS might help explain the low prescription upon discharge rates observed in our study. While only 0.01% patients were prescribed opioids for use at home in this study, Sonderman et al. [13] found that 68% of patients were prescribed them in the USA. However, a day-long stay is common for appendectomy in the USA [11], and even desired, whereas the average stay found in our study was 5 days. With a longer hospital stay, the postoperative pain can be treated while being monitored by health providers in-hospital and, once discharged the patient might no longer be experiencing pain that requires opioid use. On the other hand, while a short stay is associated with effectiveness in healthcare, pain medication for use at home will be required, which is liable to be diverted from its original purpose and abused.

Regarding the perioperative use of opioids, patients who used narcotic and non-narcotic opioids preoperatively had higher postoperative opioid use, which corroborates with previous studies [12]. This can be a result of higher pain levels both before and after surgery for those patients. Another possibility is opioid-induced hyperalgesia, when the proinflammatory property of opioids can cause higher sensitivity to pain [23], but this is more common with prolonged exposure to opioids.

Differently from before the day of surgery, the use of narcotic opioids on the day of surgery did not increase the proportion of postoperative opioid use when compared to the overall proportion. This is most likely due to over 98% of patients being administered narcotic opioids on this day and making the proportion of postoperative opioid use very similar to the overall proportion. This high opioid use on the day of surgery can be attributed to the use of opioids intraoperatively, since opioids like fentanyl and remifentanil are commonly used as part of anesthesia protocols.

The perioperative use of non-opioid analgesics had similar proportions of postoperative opioid use, except for the group administered preoperative flurbiprofen, who had higher POAP in comparison to the others. This might be related to patient’s pain levels, as flurbiprofen is a medication used for moderate pain in pediatric patients [24], although its use has been discouraged in a study by Ziesenitz et al. [25]. Finally, many patients used multiple analgesics for their perioperative pain management, so it is difficult to establish an association between specific medications and opioid use, as other medications might be confounders.

A large variation in postoperative opioid use proportion was observed among hospitals. Less than 40% of the hospitals in this study were responsible for all the postoperative opioid prescriptions, and a tendency for bigger hospitals to have higher case volumes and higher POAP was also observed. Few hospitals administered postoperative opioids to all their patients, and these facilities usually had fewer than four patients, despite having varied size classification and being in different prefectures. However, when analyzing facilities with cases < 10 and cases ≥ 10, the POAP observed was not too dissimilar between them. Facilities with cases < 10 in general were more prone to not administering opioids at all, but maintained slightly higher POAP than facilities with cases ≥ 10, which were more often prescribers of postoperative opioids. This might mean than hospitals with less cases tend not to prescribe opioids, but when they do prescribe, there is a tendency for prescribing to most patients.

This variation can be associated with a lack of standardized guidelines and evidence-based recommendations regarding postoperative opioid use in this surgery, which attributes the variations mostly to the physicians and hospital preferences and prescribing culture, which has been observed in opioid prescription variation studies before [26, 27]. Confounders regarding individual patient and disease characteristics may also contribute to this variation.

A regional variation was also observed. Although it is important to note that the sample of hospitals included in this study is not necessarily representative of each prefecture due to the structure of DPC data retrieval, it is still a relevant variation to explore in future studies. Geographical variations in opioid prescribing have been observed by other researchers [26, 28–31], but there are few established factors that can explain it. Some factors that have been associated with geographical variation of opioid prescribing are the number of available physicians [30], the distribution of pain specialists among hospitals [32], and the clinical competence and specialty of the prescribing physicians [33, 34]. Others that may be related include demographic composition such as unemployment [29], which is affected by patient education, presence of specialized teams [35, 36], and prescriber culture of the area [26].

The main limitations in this study were intrinsic to the retrospective study design and the use of DPC data. The absence of pain scores in the DPC data means we can evaluate the administration of opioids, but not the need that patients had according to their pain levels. Also, the measure of time of procedures in DPC data is in days, not hours. Thus, it is not possible to tell precisely when medications were administered in relation to surgery when it happened on the day of the surgery. Other limitations include the use of the proportion of patients who used opioids as an outcome indicator instead of morphine milligram equivalents (MME), which would be able to take dosing into consideration as well. As most of the medication administered in-hospital was intravenous, and MME is mostly for patch and oral medication, it was not possible to use it reliably. Confounders were also a barrier in establishing relations between the use of postoperative opioids and clinical and institutional factors. More research is required to properly evaluate those relationships.

Despite its limitations, our study was able to provide new information on the use of opioids for pediatric patients after appendectomy and identify related factors and geographical variations in opioid prescription in Japan. The understanding of current opioid prescribing practices can lead to the development of pediatric guidelines and evidence-based recommendations that detail the appropriate use or nonuse of opioids so we can avoid unnecessary opioid exposure in pediatric patients.
